# Comparison of hemostatic agents in patients with spontaneous intracerebral hemorrhage: A protocol for a network meta-analysis

**DOI:** 10.1097/MD.0000000000022876

**Published:** 2020-10-23

**Authors:** Yujian Li, Jun Zheng, Huiqing Zhou, Xiang Yang, Hao Li

**Affiliations:** a From the Department of Neurosurgery, West China Hospital, Sichuan, University; b From the Department of Intensive Care Unit, Fourth People's Hospital of Sichuan Province, Chengdu, P.R. China.

**Keywords:** hematoma expansion, hemostatic agents, outcome, spontaneous intracerebral haemorrhage

## Abstract

**Background:**

Spontaneous intracerebral hemorrhage (sICH) is a serious stroke subtype. The effective therapies for patients with sICH are still unclear, and the role of hemostatic agents in sICH is still unclear. Although some studies have shown that hemostatic agents could benefit patients with sICH, different hemostatic drugs have different effects on patients with sICH, and which hemostatic drug has the best effect on the prevention of hematoma expansion and neurological deterioration in sICH patients remains unclear. To better understand the effects of hemostatic agents in patients with sICH, it is necessary to carry out a network meta-analysis to comprehensively compare the effects of different hemostatic agents.

**Methods:**

This protocol has been designed following the Preferred Reporting Items for Systematic Review and Meta-Analysis Protocols statement. Related studies in the following databases will be searched until September 2020: PubMed, Embase, Scopus, Web of Science, the Cochrane Library, China National Knowledge Infrastructure, VIP and Wanfang. Randomized controlled trials and nonrandomized controlled studies comparing at least 2 different hemostatic agents in sICH patients will be included. A quality assessment will be conducted with the Cochrane Collaboration tool or the Newcastle-Ottawa Scale based on the study design. The primary outcome will be the incidence of hematoma expansion, and the secondary outcome will be the functional outcome. Pairwise and network meta-analyses will be conducted using STATA V.14 (StataCorp, College Station, Texas, USA). Mean ranks and the surface under the cumulative ranking curve will be used to evaluate every agent. Statistical inconsistency assessment, subgroup analysis, sensitivity analysis and publication bias assessment will be performed.

**Results:**

According to disseminate through academic conferences, the results of this network meta-analysis are expected to publish in a peer-reviewed journal.

**Conclusion:**

This study will provide high quality evidence about effects of different hemostatic agents in patients with sICH.

**Registration number:**

CRD42020196039.

## 1 Introduction

Spontaneous intracerebral hemorrhage (sICH) is a devastating health condition, accounting for 10% to 15% of all strokes,^[1]^ and has high mortality and morbidity and limited treatment options.^[2]^ The occurrence of hematoma expansion (HE) was detected within 3 hours of symptom onset in approximately 73% of sICH patients, clinically obvious expansion was present in 35% of patients, and HE was independently related to death and adverse outcomes.^[3–5]^

In theory, early interventions to reduce hematoma volume may improve prognosis. Compared to medical management alone, surgical craniotomy to evacuate supra tentorial hematoma and reduce hematoma volume was found to reduce mortality and disability rates, but the result is not very reliable, so surgical treatment is not often used.^[6]^ Hence, drug (ie, nonsurgical) interventions to accelerate hemostasis and limit HE have been the main focus of treatment for acute sICH.

Some studies about hemostatic agents, including aminocaproic acid, tranexamic acid, aprotinin, recombinant activated factor VII (rFVIIa), and hemocoagulase, in patients with sICH have been published previously. For example, Stephan et al randomly assigned sICH patients to receive a placebo, 20 μg of rFVIIa per kilogram of body weight, or 80 μg of rFVIIa per kilogram to compare the percentage of hematoma volume change and poor outcomes in patients from these 3 groups.^[7]^ In Sprigg et al.'s study, tranexamic acid was compared with a placebo.^[8]^ In another study by Zhang et al., hemocoagulase and tranexamic acid were analyzed.^[9]^ In addition, Piriyawat et al. explored the potential role of aminocaproic acid in the prevention of early HE after sICH,^[10]^ and aprotinin was analyzed in a study by Li.^[11]^ Some systematic reviews have also been published. In Yuan et al.'s study, rFVIIa was compared with a placebo.^[12]^ In another study by Huang et al., tranexamic acid and placebo were analyzed.^[13]^

However, different hemostatic drugs have different effects on patients with sICH, and it is still unclear which hemostatic agent is most suitable for preventing HE and neurological deterioration in sICH patients. Based on its methodology, a network meta-analysis can evaluate the relative efficiency of different hemostatic drugs and rank them.^[14]^ To better understand hemostatic treatment of sICH patients, it is necessary to carry out a network meta-analysis to comprehensively compare the effects of different hemostatic agents.

## 2 Methods

### 2.1 Study registration

This network meta-analysis protocol has been registered on the PROSPERO website (https://www.crd.york.ac.uk/prospero/) and the study registration number is CRD42020196039. The protocol has been reported following the Preferred Reporting Items for Systematic Review and Meta-Analysis Protocols^[15]^ statement (see Table, Supplemental Content 1, which illustrates the page number for the relevant items).

### 2.2 Dissemination and ethics

This study will be carried out by Bayesian network meta-analysis. This study aims to compare the efficacy of different hemostatic agents, including aminocaproic acid, tranexamic acid, aprotinin, rFVIIa, and hemocoagulase, in patients with sICH using Bayesian network meta-analysis. The final results of this study will be published in a peer-reviewed journal. Because this network meta-analysis will be based on publications, ethics approval and patient consent are not required.

### 2.3 Inclusion criteria

#### 2.3.1 Type of patients

Adult sICH patients diagnosed by CT or MRI will be included in this study. Studies on the following conditions will not be included: secondary intracerebral hemorrhage, subarachnoid hemorrhage, primary intraventricular hemorrhage or ischemic stroke.

#### 2.3.2 Type of studies

Randomized controlled trials (RCTs) and nonrandomized controlled studies will be included in this study. Case reports, case series and reviews will not be included in this study.

#### 2.3.3 Type of interventions

Studies comparing at least 2 different hemostatic agents among the following will be included in this study: aminocaproic acid, tranexamic acid, aprotinin, rFVIIa, and hemocoagulase.

#### 2.3.4 Types of outcomes

The primary outcome will be incidence of HE. HE will be evaluated by the imaging index threshold of each study. The secondary outcome will be the functional outcome at the end of follow-up. Functional outcome will be categorized as good or poor according to the scale and threshold in each study.

### 2.4 Search strategy

We will conduct the literature search for the related RCTs and nonrandomized controlled studies until September 2020 in the following databases: PubMed, Embase, Scopus, Web of Science, the Cochrane Library, China National Knowledge Infrastructure, VIP and Wanfang. Restrictions on language will not be set. The detailed search strategy is provided in online (see Table, Supplemental Content 2, which illustrates the detailed search strategy).

### 2.5 Study selection

Two authors (YL and JZ) will independently screen the titles and abstracts of all records after removing duplicates. Any record which does not meet the eligibility criteria will be deleted. The full-text papers of the remaining studies will be obtained and screened by 2 authors independently. Studies that meet the eligibility criteria will eventually be included. If data are used in more than 1 study, the study with the larger sample size and longer follow-up time will be included. Any controversy between the 2 authors will be addressed by another author (XY).

### 2.6 Data extraction

According to a predetermined extraction form, 2 authors (YL and JZ) will independently extract data from all included studies. The following information will be extracted: first author, year of publication, area, study duration, sample size, age, percentage of males, time from onset to first brain CT scan, time from first CT to follow-up CT, inclusion/exclusion criteria, details of the intervention in each group, number of patients in each group, follow-up time and outcomes in each group. We will attempt to contact authors to access data that cannot be gotten directly from the papers. Any controversy between 2 authors will be addressed by consensus, and another author (XY) will review all the data.

### 2.7 Quality assessment

The quality of all RCTs will be evaluated with the Cochrane Collaboration tool. The quality of all nonrandomized controlled studies will be assessed by the Newcastle-Ottawa Scale. Two authors (YL and JZ) will independently conduct quality evaluations, and any controversy will be addressed by discussion with another author (XY).

### 2.8 Data analysis

#### 2.8.1 Data synthesis

If quantitative analysis cannot be carried out, the results will be described narratively. When quantitative analysis is feasible, we will conduct all of the following statistical analyses by STATA V.14 (StataCorp, College Station, Texas, USA).

#### 2.8.2 Direct comparisons of interventions

If at least 2 studies provide related data, conventional pairwise meta-analyses between different interventions will be performed first using the DerSimonian–Laird method and a random effects model.^[16]^ Heterogeneity among the included studies will be assessed by the I^2^ statistic.^[17]^

#### 2.8.3 Indirect and mixed comparisons of interventions

A network meta-analysis will be conducted using a random effects model.^[18]^ The network geometry will indicate the interactions among all the included studies, and the contributions of direct comparisons will be shown in the contribution plot for the network.^[19]^ The effects of every intervention on both the incidence of HE and the functional outcome in sICH patients will be assessed using mean ranks and the surface under the cumulative ranking curve.^[20]^

#### 2.8.4 Statistical inconsistency assessment

Inconsistency between direct and indirect comparisons will be evaluated using global and local methods. The design-by-treatment model will be adopted for the global method,^[21]^ and the local inconsistency will be assessed using the loop-specific method.^[22]^

#### 2.8.5 Subgroup analysis and sensitivity analysis

We will perform subgroup analyses, if possible, according to age, sex, race, Glasgow Coma Scale score, baseline hematoma volume and hematoma location. Sensitivity analysis, by eliminating each study, will be used to test whether the results are stable.

#### 2.8.6 Publication bias

The network funnel plot will be used to assess the potential publication bias in the network meta-analysis.

#### 2.8.7 Quality of evidence

We will follow the Grading of Recommendations, Assessment, Development and Evaluation approach, which is used to rate the quality of treatment effect evaluations from network meta-analyses, to assess the evidence quality.^[23]^

## 3 Discussion

This study will be the first network meta-analysis that comprehensively compares different hemostatic agents in sICH patients. Nonrandomized controlled studies will be included to strengthen the statistical power of this network meta-analysis because of the limited number of related randomized controlled studies. We hope that the results of this network meta-analysis will offer more information about the safety and efficacy of different hemostatic agents in sICH patients and provide help for future clinical practice and research design. However, this network meta-analysis will still have limitations. First, studies with inferior quality that are included in this network meta-analysis may decrease the significance of the results. Moreover, the final results of this network meta-analysis may be influenced by high heterogeneity among different studies.

In conclusion, this study will help to compare the effects of different hemostatic agents in patients with sICH. We hope this network meta-analysis can offer a high evidence for hemostatic treatment of sICH patients and guide future clinical practice.

## Author contributions

**Conceptualization:** Yujian Li, Hao Li.

**Data curation:** Yujian Li, Huiqing Zhou, Xiang Yang.

**Methodology:** Jun Zheng.

**Project administration:** Xiang Yang.

**Writing – original draft:** Yujian Li.

**Writing – review & editing:** Hao Li.

## Supplementary Material

Supplemental Digital Content

## Supplementary Material

Supplemental Digital Content
