# Supplementary material for: Comparison of hemostatic agents in patients with spontaneous intracerebral hemorrhage: A protocol for a network meta-analysis
Source: Medicine (Baltimore). 2020 Oct 23;99(43):e22876. doi: 10.1097/MD.0000000000022876 (PMC7581098; doi:10.1097/MD.0000000000022876)
Supplement: Supplemental Digital Content [file medi-99-e22876-s002.docx]

**Supplement 2** Search strategy

| #1 | “Cerebral Hemorrhage” [Mesh] OR “Intracranial Hemorrhage, |
| --- | --- |
|  | Hypertensive” [Mesh] |
| #2 | Hemorrhagic stroke OR Haemorrhagic stroke |
| #3 | Intracerebral OR Cerebral OR Brain OR Intracranial OR |
|  | Intraparenchymal OR Putaminal OR Basal ganglia OR Thalamic |
| #4 | Hemorrhage OR Haemorrhage OR Hematoma OR Haematoma OR |
|  | Bleeding |
| #5 | “Hemostatic” [Mesh] OR “Styptic” [Mesh] OR “Anastaltic” [Mesh] OR “Hemostyptic” [Mesh] |
|  |  |
|  |  |
| #6 | Hemostatic agents OR Hemostatic drugs OR Aminocaproic acid OR Tranexamic acid OR Aprotinin OR Recombinant activated factor VII OR Hemocoagulase |
|  |  |
|  |  |
| #7 | #1 OR #2 OR (#3 AND #4) |
| #8 | #5 OR #6 |
| #9 | #7 AND #8 |
